# Protective effects of a novel FS-Collagen hydrolysates against UV- and d-galactose-induced skin aging

**DOI:** 10.1007/s10068-024-01660-7

**Published:** 2024-08-07

**Authors:** Yihan Zhang, Guangye Huang, Zhen Zhang, Yongzhao Xu, Jiaoyan Ren, Wei Sun, Jianwen Chen, Ruikun He

**Affiliations:** 1 School of Biology and Biological Engineering, South China University of Technology, Guangzhou Higher Education Mega Center, Panyu District, Guangzhou, China; 2 School of Pharmaceutical Sciences, Sun Yat-Sen University, Guangzhou Higher Education Mega Center, Panyu District, Guangzhou, China; 3 BYHEALTH Institute of Nutrition & Health, Kexue Avenue Central, Huangpu District, Guangzhou, China; 4 School of Food Sciences and Engineering, South China University of Technology, Guangzhou Higher Education Mega Center, Panyu District, Guangzhou, China; 5 Guangdong Provincial Key Laboratory of Chiral Molecule and Drug Discovery, Guangzhou Higher Education Mega Center, Panyu District, Guangzhou, China

**Keywords:** Collagen hydrolysates, Skin aging, Collagen I, Hyaluronic acid, Cellular connections

## Abstract

The oral consumption of collagen hydrolysates derived from various animal tissues has been demonstrated to have beneficial effects on skin health, particularly in combating the signs of aging. Here in this study, a novel animal-derived FS-Collagen hydrolysates were developed and its effects against skin aging was analyzed in a new mice skin aging model established through a combination of UV irradiation and d-galactose induction. 8 Weeks of oral FS-Collagen administration demonstrated significant protective effects against skin aging in mice, evidenced by the preservation of the skin’s macroscopic appearance, the restoration of skin composition and structure, an enhancement in antioxidant capacity and the inhibition of inflammation. Additionally, FS-Collagen safeguards skin barrier integrity and cellular connections, particularly by maintaining the expression levels of Dsg1 and Jam-A. In summary, the FS-Collagen exhibits positive effects in countering skin aging and holds promise as an alternative functional nutrition supplement for anti-skin aging care.

**Supplementary Information:**

The online version contains supplementary material available at 10.1007/s10068-024-01660-7.

## Introduction

Skin is the largest organ of the body and serves as its primary protective barrier. The intricate structure of skin confers its ability to maintain an internal environment while protecting against external damages (Arda et al., 2014). Histologically, our skin is comprised of three primary layers, epidermis, dermis, and subcutaneous fat. Each of these layers consists of different cell types and achieves various biological functions (Gould, 2018).

Skin aging is a topic of great interest due to its both psychological and physical impacts, primarily because of its visibility. This aging process of the skin is multifaceted, affecting nearly every layer and function of the skin. The intrinsic factors of skin aging encompass chronological-based physiological alterations, genetic factors and hormones, which is predominantly manifested as fine lines, xerosis (dry skin) and laxity (Langton et al., 2012). On the other hand, extrinsic aging, predominantly induced by environmental factors such as air pollution, UV irradiation, and smoking, usually leads to coarse wrinkles, irregular pigmentation, lentigines and age spots (Csekes and Račková, 2021). The study of skin aging has delved deeply into its molecular mechanisms, like the oxidative stress, loss of collagen and elastin, the alterations of cell connections (Hensley and Floyd, 2002; Kirschner and Brandner, 2012; Zorina et al., 2022) and the IL-17-expressing T helper cells-mediated chronic inflammation skin aging (Solá et al., 2023), paving the way for potential interventions to decelerate this process.

To date, several clinical and nutritional treatments have been devised to combat skin aging. The nutritional administrations, with their inherent safety and comprehensive effects, have gained prominence for addressing skin aging concerns. Functional foods like dietary plant extract supplements, which are rich in natural polyphenols, and animal-derived collagen peptides are now with the most attention (Domaszewska Szostek et al., 2021; Kong et al., 2018). The collagen derived from various animals has been demonstrated with a protective effect on skin, and some underlying molecular mechanisms have also been unveiled (Kang et al., 2021; Zhuang et al., 2009).

In this study, we developed a novel collagen named FS-Collagen (Hgallagen®) by previous work (Sun et al., 2023), which is derived from Atlantic cod fish (*Gadus morhua*) skin and chicken sternal cartilage. The FS-Collagen is rich of glycosaminoglycans (GAGs) at a content of 4.2%. The GAGs is reported to be very important in the skin rejuvenation (Salbach et al., 2012), but is rarely concerned in the existing collagen hydrolysates development. In this study, we comprehensively evaluate the anti-aging effect of this GAGs-enriched FS-Collagen on the mice skin. Aiming to simulate human aging in a real-world context, we introduced a novel skin aging model induced by the combination of d-galactose and UV, mimicking both the intrinsic and external risk factors. The performance of the FS-Collagen on this special model, demonstrated its effects on maintaining the macroscopic appearance, elasticity, hydration, barrier integrity, and histologically structure of the skin. FS-Collagen demonstrated bioactivities in maintaining the collagen and hyaluronic acid levels, as well as the capacity of antioxidation and anti-inflammation. Moreover, it showed positive effects on maintaining the cell connections, which contributing to the skin integrity and hydration. Together, these data suggest that FS-Collagen may serve as a promising functional food supplement for promoting skin health.

## Materials and methods

### Preparation of FS-Collagen

FS-Collagen was prepared following established procedures (Sun et al., 2023). Briefly, cod fish skin and chicken sternal cartilage were first cleaned and then pulverized into coarse particles. The resulting mixture was thoroughly blended and subsequently subjected to a 30 min heating process at 100 °C, followed by enzymatic digestion for 60 min. After enzymolysis, the mixture was heated again at 100 °C for 10 min, and then carried out the filtration, concentration and drying process.

### Characterisation of FS-Collagen

The amino acid composition of the FS-Collagen was determined by the HPLC/MS (AB Sciex, American), following the established procedure (Sun et al., 2023).

The molecular weight of the peptides was measured using Shimadzu LC-20A HPLC system (Agilent Technologies, Inc., Santa Clara, California, USA) with GPC column (TSK G2000 SWXL, 7.8 × 300 mm, particle size 7 μm, Tosoh, Tokyo, Japan). The column was equilibrated with acetonitrile/water/trifluoroacetic acid (45/55/0.1, v/v/v). Samples were applied to the column, eluted at a flow rate of 0.5 mL/min and monitored at 214 nm at 30 °C. A molecular weight calibration curve was prepared from the average retention times of the following standards: bovine serum albumin (66,430 Da), cytochrome C (12,400 Da), aprotinin (6511 Da), bacitracin (1423 Da), glutathione (307 Da) and glycylglycine (132 Da). All these standards were obtained from Sigma-Aldrich (St. Louis, MO, USA).

The glycosaminoglycans (GAGs) were determined by an uronic acid carbazole reaction method (Bitter and Muir, 1962). And the sodium hyaluronate in the sample was enzymatically hydrolyzed into sodium hyaluronate disaccharide, and determined by high-performance liquid chromatography.

### Animals and treatment

BALB/c mice (male, 7–8 weeks old) were obtained from Guangdong Medical Laboratory Animal Center (Guangzhou, China) and acclimated to the housing conditions for one week with access to food and water ad libitum for one week before the FS-Collagen treatment. Subsequently, the mice were randomly divided into five groups, each consisting of 10 mice: the control group (Ctrl), the model group (Mdl), and the FS-Collagen treatment groups at the dosages of 0.5 g/kg (L), 1 g/kg (M), and 2 g/kg (H). All mice underwent a 2 cm * 3 cm dehairing at the back skin by shaving and hair removal cream every 2 or 3 days.

To induce skin aging, groups Mdl, L, M, H were injected subcutaneously with a daily dose of 1.0 g/kg BW of d-galactose solution at the back of mice neck and exposed to UV irradiation 6 days a week for 8 weeks. The UV irradiation was carried out using an UV machine (FBS-OUV1, Fubusi, Xiamen, China) with UVA wavelength at 340 nm and UVB wavelength at 313 nm. The distance between the lamp and the back of the mice was 30 cm, and each irradiation session lasted for 2 h each time. The control group (Ctrl) received an equivalent volume of saline injection and did not undergo UV irradiation.

The mice received oral daily administration of FS-Collagen or distilled water via gavage before the d-gal and UV treatment. The skin elasticity and hydration were evaluated at the 0, 4, and 8 weeks into the administration. After the last irradiation, the mice were anesthetized and the tissues were sampled for subsequent analysis. All animal experiments were conducted in accordance with the recommendations of the Animal Care and Use Committee at Sun Yat-sen University (Guangzhou, China).

### Imaging of skin macroscopic appearance and wrinkle evaluation

The skin macroscopic appearance changes were imaged at 0, 4th, and 8th week post FS-Collagen intervention. The wrinkles and erythema on the skin were observed. The assessment of wrinkles followed the procedures outlined by Yoshimura et al. (2021). The wrinkles were evaluated based on the following criteria: Grade 0, fine striations across the back, visible and disappearing with motion; Grade 1, a few shallow, coarse wrinkles across the back, visible and disappearing with motion; Grade 2, some coarse wrinkles across the back that are consistently present; Grade 3, several deep, coarse wrinkles across the back that are consistently present.

### Measurement of skin moisture content, transepidermal water loss and elasticity

The skin moisture content and transepidermal water loss were measured by AS-VT100RS (ASCH, Japan) and HKJ-SK-03 skin tester (Hunkey, Japan) respectively, according to the manufacturer’s instructions. The skin elasticity was measured by a Cutometer MPA 580 (Courage and Khazaka Electronics, Cologne, Germany). Each measurement was performed in triplicate.

### H&E and Masson’s trichrome staining

Skin tissues of mice were dissected, fixed in 10% phosphate-buffered formalin, embedded into paraffin, and sectioned into 5 µm slices. These deparaffinized skin sections were stained with H&E and Masson’s trichrome to evaluate histological structure and collagen levels. The stained region was photographed using an Eclipse Ti-S inverted fluorescence microscope (Nikon, Japan), and the dermal collagen density was measured and analyzed.

### Measurement of skin component content

The content of collagen I (Mouse Collagen Type I ELISA Kit, CUSABIO), collagen III (Mouse Collagen Type III ELISA Kit, CUSABIO), hydroxyproline [Hydroxyproline (HYP) Content Assay Kit, BC0250, Solarbio] and hyaluronic acid (Hyaluronan Elisa Kit, SEKH-0509, Solarbio) were measured by the corresponding testing kits.

### Determination of oxidation and inflammation associated biomarkers

The oxidation levels were measured by the Lipid Peroxidation MDA Assay Kit (S0131, Beyotime), Reactive Oxygen Species Assay Kit (S0033, Beyotime), Total Superoxide Dismutase Assay Kit with WST-8 (S0101, Beyotime), and Total Glutathione Peroxidase Assay Kit with NADPH (S0058, Beyotime), according to the manufacturer’s instructions.

The inflammation markers IL-1β, IL-6, CXCl8, and TNF-α were measured by corresponding ELISA kits (SEKM-0002, SEKM-0007, SEKM-0046, and SEKM-0034, Solarbio).

### Immunohistochemical staining and western blotting

The impact of FS-Collagen on cell junctions and tissue components was determined through immunohistochemical analysis, focusing on marker genes associated with various types of cell junctions and key skin tissue components such as collagen I, Filaggrin, and Aquaporin 3. Mouse skin tissues were embedded in paraffin and sectioned into 5 µm slices. Immunohistochemical staining was carried out and primary antibody against Desmoglein-1 (Anti-Desmoglein 1/DSG1 antibody, Abcam, 1:250), Claudin-1 (Anti-Claudin-1 Rabbit pAb, Servicebio, 1:400), ZO-1 (Anti-ZO-1 tight junction protein Rabbit pAb, Servicebio, 1:600), E-Cadherin (Anti-E-Cadherin Mouse mAb, Servicebio, 1:1000), JAM-A (AM-A Rabbit mAb, Cell Signaling Technology,1:50), Collagen I (Anti-Collagen I Rabbit pAb, Servicebio, 1:1000), Aquaporin 3 (Anti-Aquaporin 3 Rabbit pAb, Servicebio, 1:1000), Filaggrin (Anti-Filaggrin antibody, Abcam, 1:500), NRF2 (Anti-NRF2 Rabbit pAb, Servicebio, 1:1000), HO-1 (Anti-Heme Oxygenase 1 Rabbit pAb, Servicebio, 1:500) were used.

Western blotting analysis was carried out according to the standard protocol. Primary antibodies against GAPDH (GAPDH Rabbit mAb, Cell Signaling Technology), α-Tubulin (α-Tubulin Rabbit mAb, Cell Signaling Technology), MMP-1(MMP-1 Rabbit mAb, Cell Signaling Technology), Hyaluronan synthase 2 (Anti-Hyaluronan synthase 2 antibody, Abcam), HYAL1 (Anti-HYAL1 antibody, Abcam), β-Galactosidase (β-Galactosidase Rabbit mAb, Cell Signaling Technology), p21 (p21 Rabbit mAb, Cell Signaling Technology), p53 (p53 Rabbit mAb, Cell Signaling Technology), and JAM-A (JAM-A Rabbit mAb, Cell Signaling Technology) were used.

### Statistics

The data are presented as the mean ± s.e.m. And the one-way ANOVA/Bonferroni test or Dunnett’s T3-test was used to determine the differences between the groups. *p* value less than 0.05 was determined to be statistically significant.

## Results and discussion

### Characterization of FS-Collagen hydrolysates

#### Amino acid composition

The amino acid composition of FS-Collagen is depicted in Table 1. Glycine stands out as the most prevalent amino acid in the sample, a characteristic feature of collagen that distinguishes it from other proteins. Apart from glycine, proline and hydroxyproline, the other two amino acids that constitute the distinctive Gly-X-Y tripeptides in collagen, are also notably abundant in FS-Collagen. Particularly noteworthy is glutamine, which ranks as the second most abundant amino acid in the FS-Collagen.

**Table 1:** The amino acid composition of FS-Collagen

Amino acid types	Content (mg/g)
Gly	194.52
Glu	95.67
Pro	86.16
Hyp	77
Ala	74.93
Arg	74.61
Asp	59.81
Ser	42.3
Lys	38.03
Leu	26.5
Thr	23.89
Val	19.03
Ile	14.11
Phe	14.03
Met	13.83
His	9.46
Tyr	5.54

#### Peptide molecular weight distribution

**Fig. 1:**
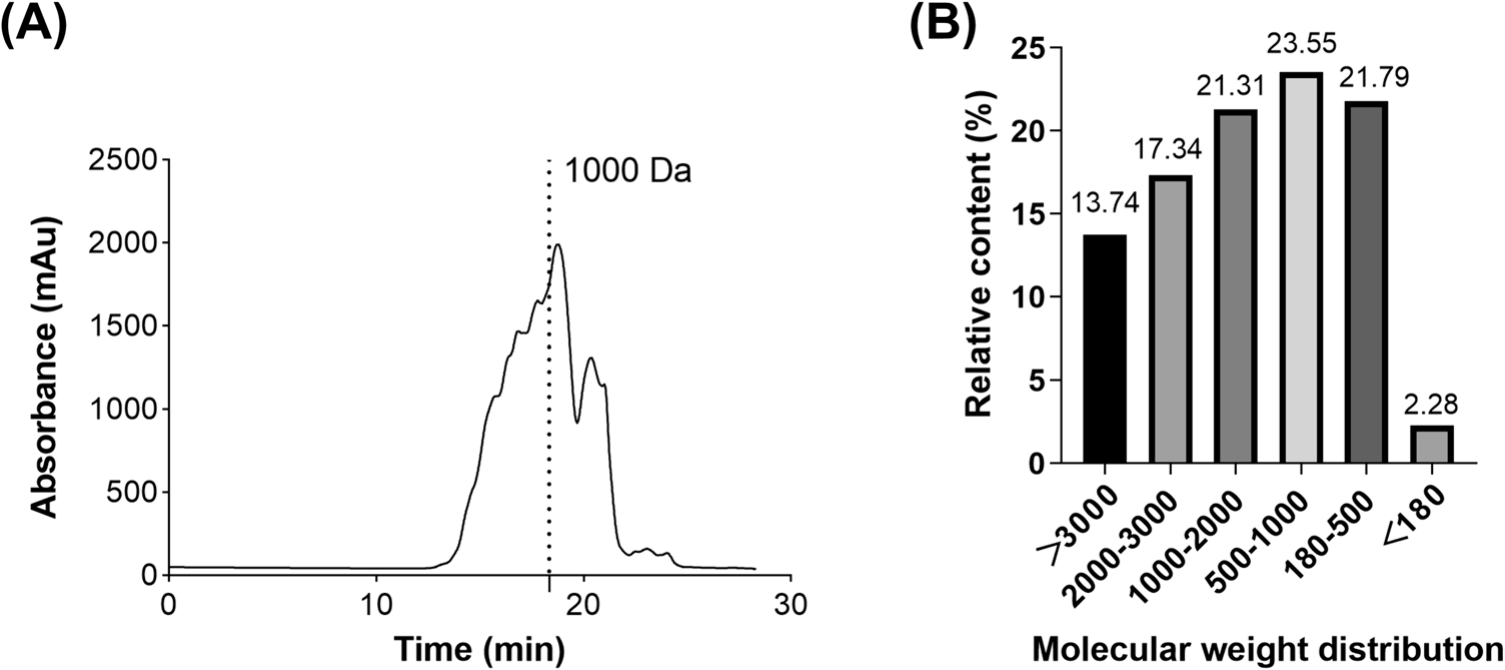
FS-Collagen peptide molecular weight distribution. (**A**) Absorbance pattern of the FS-Collagen. (**B**) Peptides molecular weight distribution

The peptide molecular weight distribution was determined by an HPLC/MS system. As shown in Fig. 1(B), over 47% of the peptides had a molecular weight of less than 1000 Da.

#### GAGs content

The glycosaminoglycans (GAGs) content in FS-Collagen hydrolysates is 4.2% (expressed as hyaluronic acid).

In this study, we have developed a novel collagen formulation, FS-Collagen, by extracting collagen hydrolysates from Atlantic cod fish (*G. morhua*) skin and chicken sternal cartilage mixture. Traditionally, collagens are rich in glycine, proline and hydroxyproline, essential amino acids critical for skin health, and dietary supplements of these amino acids can compensate for the inadequate endogenous synthesis (Li and Wu, 2018). Besides, the collagen hydrolysates can be absorbed as dipeptide forms, like Gly-Pro and Pro-Hyp, to exert beneficial effects on skin health (Lee et al., 2019). The new developed FS-Collagen hydrolysates in this study possess high glycine, proline and hydroxyproline content, supporting the synthesis of collagen in skin. More importantly, FS-Collagen is rich in glycosaminoglycans (GAGs), which are reported to be important for the skin regeneration (Salbach et al., 2012). The hyaluronic acid, a kind of GAGs, has also been employed as one of the imperative components of cosmetic and nutricosmetic products, due to its regenerative properties (Bukhari et al., 2018). Beyond wound healing acceleration, hyaluronic acid moisturizes dry skin and even treats psoriasis, and increases skin density and thickness (Castrejón Comas et al., 2023). Clinical studies have validated the wrinkles-relieving and dry skin-improving effects of oral hyaluronic acid intake (Michelotti et al., 2021). By supplementing deficient GAGs in aging skin, the administration of FS-Collagen may directly enhance skin hydration and elasticity. Our research not only introduces a novel collagen formulation but also emphasizes its potential to revolutionize skin health and rejuvenation.

### FS-Collagen prevents UV- and d-gal-induced macroscopic appearance changes, elasticity loss and dehydration in mice skin

To simulate the multiple factors contributing to skin aging, we combined d-galactose and UV-irradiation to induce both endogenous and exogenous aging-drivers simultaneously. d-galactose is commonly employed in numerous research studies to replicate the internal conditions associated with natural aging. The administration of d-galactose can artificially induce senescence both in vitro and in vivo, exhibiting many similarities to the natural aging process. This d-galactose-induced aging model is commonly employed in studies investigating anti-aging therapeutic interventions. Notably, it can induce substantial oxidative stress, leading to cellular and tissue damage reminiscent of the aging process. Additionally, UV-irradiation stands out as the prevailing method used to simulate the photoaging process of human skin. With this mice model, we evaluated the anti-skin aging effects of our FS-Collagen.

**Fig. 2:**
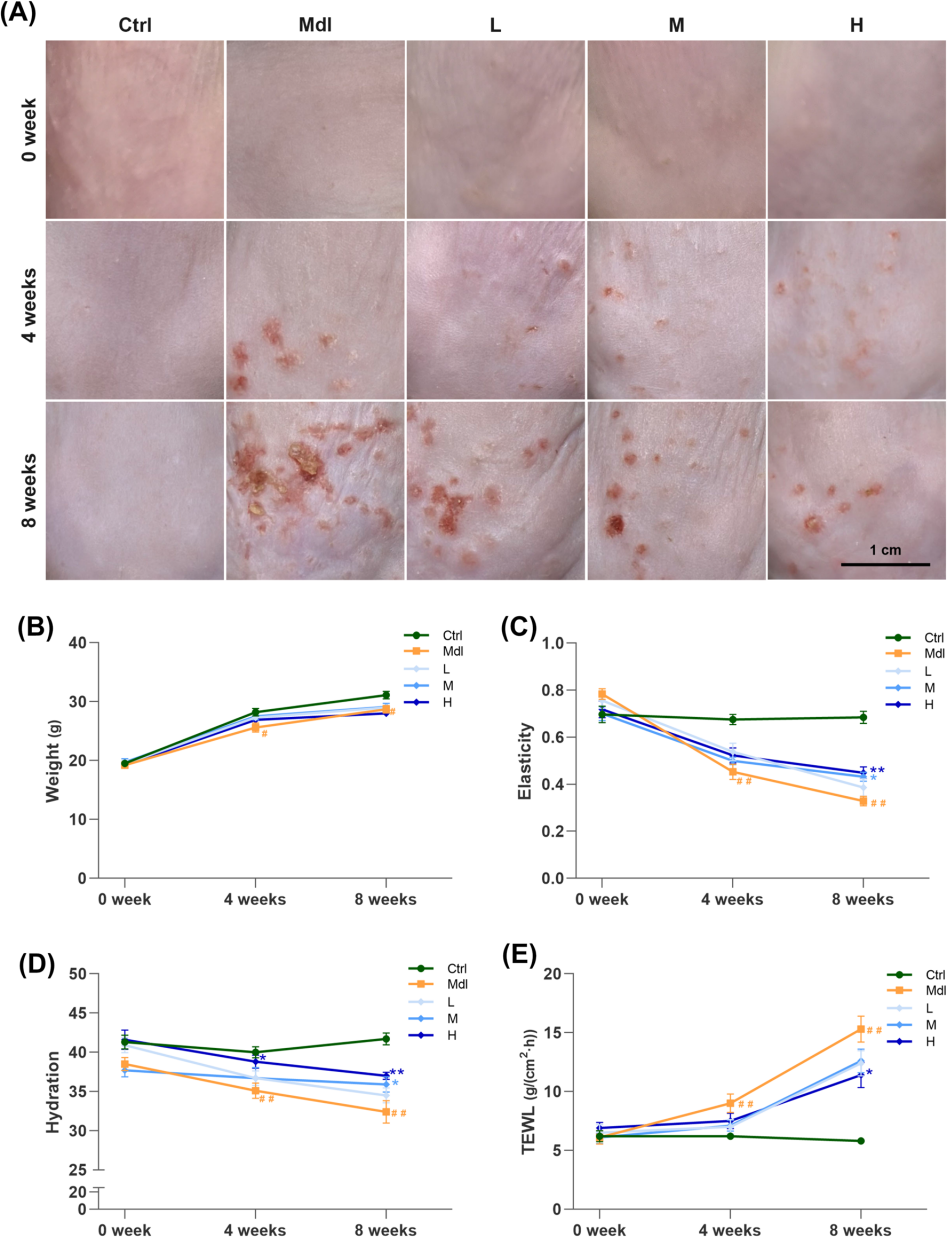
FS-Collagen peptide intervention ameliorates UV- and d-gal-induced macroscopic appearance changes, elasticity loss and dehydration. (**A**) Macroscopic appearance of the back skin of the mice. (**B**) Body weight. (**C**) Elasticity of the irradiated skin. (**D**) Skin moisture content. (**E**) Transepidermal water loss (TEWL). ^#^*p* < 0.05 compared to Ctrl; ^##^*p* < 0.01 compared to Ctrl; *p < 0.05 compared to Mdl; **p < 0.01 compared to Mdl. n = 10 in each group

The 8-week UV irradiation combined with d-galactose treatment would induce skin aging, as manifested by significant changes on the macroscopic appearance of the mice dorsal skin, including wrinkles, dryness, roughness, sagging, thickening and even erythema in Mdl group. However, FS-Collagen intervention ameliorated all of these phenotypes, even in the low dosage group [0.5 g/kg, L, Fig. 2(A)]. Moreover, no noticeable side effect on body weight lost was observed with FS-Collagen intervention [Fig. 2(B)]. The application of FS-Collagen at middle (1 g/kg) or high (2 g/kg) dosages notably preserved the skin's smoothness, elasticity, and hydration. Specifically, the skin elasticity of mice in model group showed a remarkable decrease by 52.4% when compared to the control group, whereas in 1 g/kg and 2 g/kg FS-Collagen -administration groups, the reduction was limited to 36.9% and 36.8% respectively [Fig. 2(C)]. A reduction of wrinkle formation was also observed in the high dosage group after 8 weeks of invention, comparing to that in the model group [Fig. S1(A)]. In terms of skin hydration, the FS-Collagen administration preserved the stratum corneum water content as high as 88% to that of the control group, while in the model group, it dropped to 79.4% [Fig. 2(D)]. Additionally, the transepidermal water loss (TEWL) was significantly lower in the FS-Collagen—administration group than that in the model group [Fig. 2(E)]. The erythema was observed in the UV and d-gal treated mice, probably due to the exacerbation of the aging phenotype by the combination of these two aging factors. However, the FS-Collagen exhibited the positive effects on the erythema prevention, supporting its protective effects on skin. Further optimization of this model would help to enhance its ability to simulate human aging in a more realistic context.

### FS-Collagen reduces UV- and d-gal-induced epidermal hyperplasia and dermal fibrils disruption

**Fig. 3:**
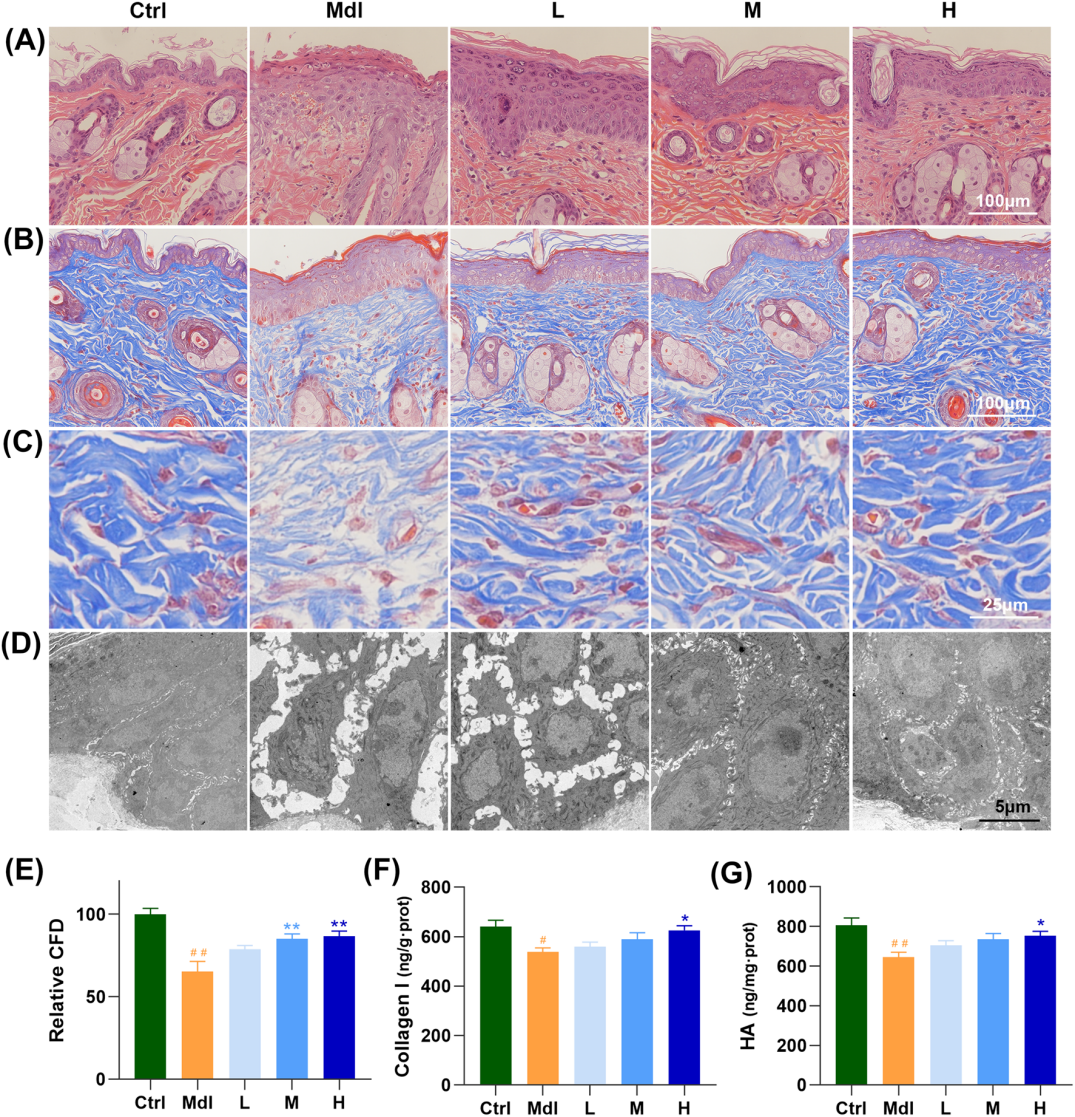
FS-Collagen peptide ameliorates UV- and d-gal-induced epidermal hyperplasia, and retains the component mass. (**A**) H&E staining of the skin tissue. (**B**) Masson staining of collagen fibrils. (**C**) Enlarged view of the collagen fibrils staining. (**D**) Transmission electron microscope imaging of skin tissue. (**E**) Relative collagen fibrils density. (**F**) Collagen I content in the skin tissue. (**G**) Hyaluronic acid content in the skin tissue. n = 5 in each group. ^#^*p* < 0.05 compared to Ctrl; ^##^*p* < 0.01 compared to Ctrl; *p < 0.05 compared to Mdl; **p < 0.01 compared to Mdl

Next, we sought to investigate how the FS-Collagen act to maintain the skin appearance, elasticity and hydration. At first, the effect of FS-Collagen on UV- and d-gal-induced damage, characterized by epidermal hyperplasia and dermal fibrils disruption, was firstly determined by the histological analysis. The model group displayed a thickened epidermis compared to the control group, while FS-Collagen intervention mitigated these pathological changes [Fig. 3(A)]. Besides, Masson staining revealed that the fibrils maintained a nearly regular content and arrangement in the FS-Collagen group, whereas the model group exhibited severe breakage and loss of collagen, and disrupting the fiber arrangement significantly [Fig. 3(B), (C), (E)]. These changes were further verified by transmission electron microscope imaging, and notably, the cells were closely arranged in order in the Ctrl group, but the Mdl group was shown with enlarged cells, loose cell–cell junctions, increased intercellular spacing and altered cell arrangements [Fig. 3(D)].

### FS-Collagen reduces UV- and d-gal-induced collagen disruption and hyaluronic acid loss

**Fig. 4:**
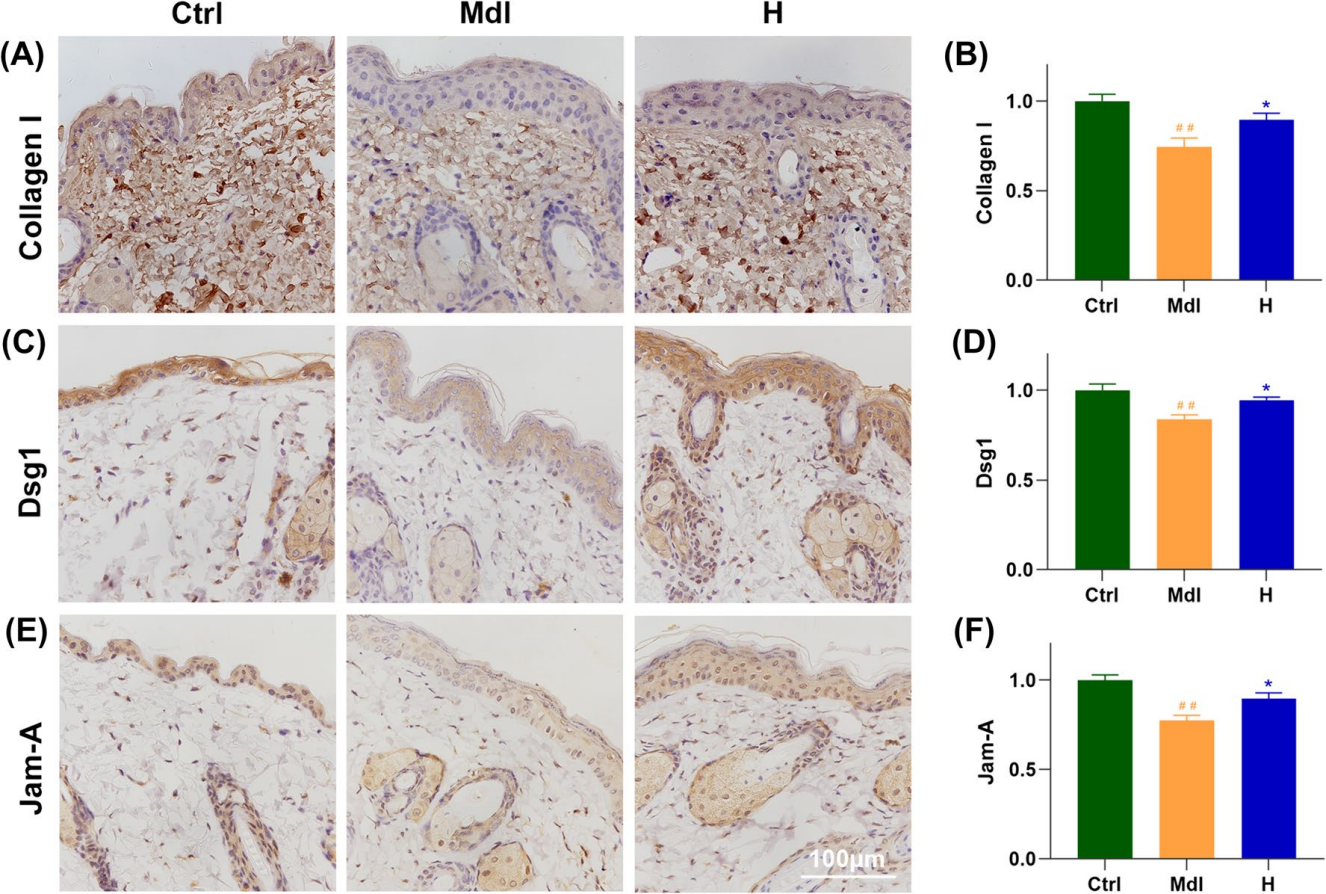
FS-Collagen retains the collagen levels and the cell junctions. (**A**, **B**) Immunochemical staining and quantification of collagen I. (**C**, **D**) Immunochemical staining and quantification of Dsg1. (**E**, **F**) Immunochemical staining and quantification of Jam-A. n = 5 in each group. ^##^*p* < 0.01 compared to Ctrl; *p < 0.05 compared to Mdl; **p < 0.01 compared to Mdl

In the realm of skin elasticity, the dermis extracellular matrix (ECM), a sophisticated blend of type I and III collagen fibrils, elastic fibers and microfibrils enmeshed in a ground substance of proteoglycans, largely dictates dermal properties (Krieg and Aumailley, 2011). Previous studies have meticulously documented the impacts of various collagen types on ECM remodeling, encompassing stimulation of collagen synthesis, inhibition of collagen degradation, and augmentation increasing elastin and hyaluronic acid content and so on (Wang et al., 2017). Therefore, we measured the content of type I and type III collagen in the mice’s skin. As shown in Figs. 3(E), (F) and 4(A), (B), FS-Collagen administration resulted in significantly elevated collagen I content compared to the model group, while collagen III only showed an increased tendency, which didn’t reach a statistical significance [Fig. S1(B)]. Similar trends were also observed in the levels of hydroxyproline, as indicated in Fig. S1(C).

We further measured the serum levels of procollagen type 1 carboxy-terminal propeptide (PICP), procollagen type 1 amino-terminal propeptide (PINP), carboxy-terminal telopeptide of type 1 collagen (CTX-1) and amino-terminal telopeptide of type 1 collagen (NTX-1) using ELISA respectively, to investigate whether FS-Collagen intervention would affect collagen I turnover, however, the results didn’t reveal any significant difference (data not shown).

The expression levels of matrix metalloproteinases and their inhibitors or activators were also detected by western blotting. As shown in Fig. S1(D), (E), FS-Collagen prevented the matrix degradation, possibly by downregulating Mmp1 expression but not by upregulating Timp2, as the expression level of Timp2 was not changed.

Besides collagen, hyaluronic acid is another crucial component of skin tissue, which plays critical roles in skin hydration, elasticity and repair. The HA content compromised with UV and d-gal treatment, but the level was rescued by FS-Collagen administration [Fig. 3(G)]. And notably, the expression levels of Hags2 and Hyal didn’t change with intervention, as shown in Fig. S1(E), suggesting the abundant GAGs in FS-Collagen may directly enhance HA supplement and contribute to skin hydration and elasticity.

Skin moisturization is determined by not only the natural moisturizing factor (NMF) found in corneocytes, cornified envelope and the well-organized stratum corneum lipids barrier, but also the regulated water transport. The NMF declines with age in elderly skin, leading to a reduction in soluble NMF in dry skin. Hyaluronic acid, a major dermal component, is naturally presents in the epidermis and regulates keratinocyte differentiation by binding to CD44 receptors (Sakai et al., 2000). Aquaporins (AQPs) constitute a family of transmembrane proteins that create specialized water channels and facilitate water flux across the cell plasma membrane. Among them, AQP-3, found in the human epidermis, plays a crucial role in aligning with the distribution of water within the epidermal layers (Sebastian et al., 2015). As reported elsewhere, oral intake of collagen hydrolysates can modulate filaggrin and Aqp3 expression to retain skin moisture (Kang et al., 2021). To investigate how the FS-Collagen administration slows down the dehydration, we further analyzed the expression pattern of the Filaggrin, which acts as the most important source of natural moisturizing factors. However, the downregulated Flg1 expression by UV- and d-gal treatment could not be rescued by FS-Collagen (Fig. S2). Moreover, the Aqp3 expression, which has been reported to be triggered by other collagen, didn’t show any obvious changes, although our previous study with human keratinocytes confirmed the effects of FS-Collagen to induce Flg-1 and AQP3 levels (Sun et al., 2023).

### FS-Collagen maintains the cell junctions

Apart from AQPs, intercellular junctions, like desmosomes and tight junctions, sealing the space between individual cells in the epidermis, are also essential for skin permeability and barrier integrity maintenances (Niessen, 2007). Desmosomes, essential structures connecting epidermal keratinocytes, provide intercellular adhesive properties, and they can transform into corneodesmosomes to provide stronger intercellular adhesion when keratinocytes differentiate into cornified cells (Évora et al., 2021). In recent years, more and more studies have revealed the presence and significance of tight junctions in the epidermis (Brandner et al., 2006). Notably, UV exposure has been demonstrated to disrupt cell cohesion and mechanical integrity, leading to accelerated skin aging.

Therefore, we sought to determine whether FS-Collagen could retain skin hydration by repairing the skin barrier, particularly the keratinocyte cell connections. We accessed the expression patterns of key proteins of different types of cell connections, including tight junction protein Zo-1 (Zo-1), claudin-1 (Cldn1) and junctional adhesion molecule A (Jam-A) for tight junctions, E-cadherin for adherens junctions, and desmoglein 1 (Dsg1) for epithelial specific desmosomes, by immunohistochemistry. Significant alterations were observed in the expression patterns of Dsg1 and Jam-A in epidermal region of the skin in model group compared to that in control group. Remarkably, FS-Collagen treatment successfully restored these phenotypes [Fig. 4(C)–(F)]. In contrast, the expression of other genes exhibited only slight changes when exposed to UV- and d-gal treatments (Fig. S2). These results suggest that the FS-Collagen may help to support the skin integrity specifically by its role in maintaining the tight junctions and desmosomes formation in epidermis.

As suggested by many literatures, modulating cell junctions could be an alternative way for skin moisturizing care. FS-Collagen administration did support the expression patterns of the key desmosomes and tight junction proteins, Dsg1 and Jam-A. Dsg1 (desmoglein 1), is a member of desmoglein family, which are a group of transmembrane glycoproteins in the desmosome complex. The desmosome, a vital structure ensuring robust cell–cell adhesion in epithelial tissues, is particularly crucial in skin integrity. Within the skin, Dsg1 assumes a pivotal role in keratinocyte adhesion, essential for upholding the structural integrity of the epidermis (Garrod and Chidgey, 2008). Jam-A (Junctional adhesion molecule-A), belonging to the immunoglobulin superfamily, is instrumental in forming tight junctions among endothelial and epithelial cells, thereby safeguarding the skin’s tight junction integrity and regulating paracellular permeability (Brandner et al., 2006). In this study, disruption of cell–cell connections was evident in the model group, as confirmed by transmission electron microscope imaging and immunochemical staining of Dsg1 and Jam-A. Administration of FS-Collagen preserved these intercellular connections, showcasing its potential in protecting the skin barrier by maintaining proper cell junctions. This effectiveness is seldom documented with other collagen hydrolysates in the existing literature, indicating a need for increased focus on the benefits and applications of collagen hydrolysates in this context.

### FS-Collagen alleviates UV- and d-gal-induced oxidation, inflammation and cellular senescence

**Fig. 5:**
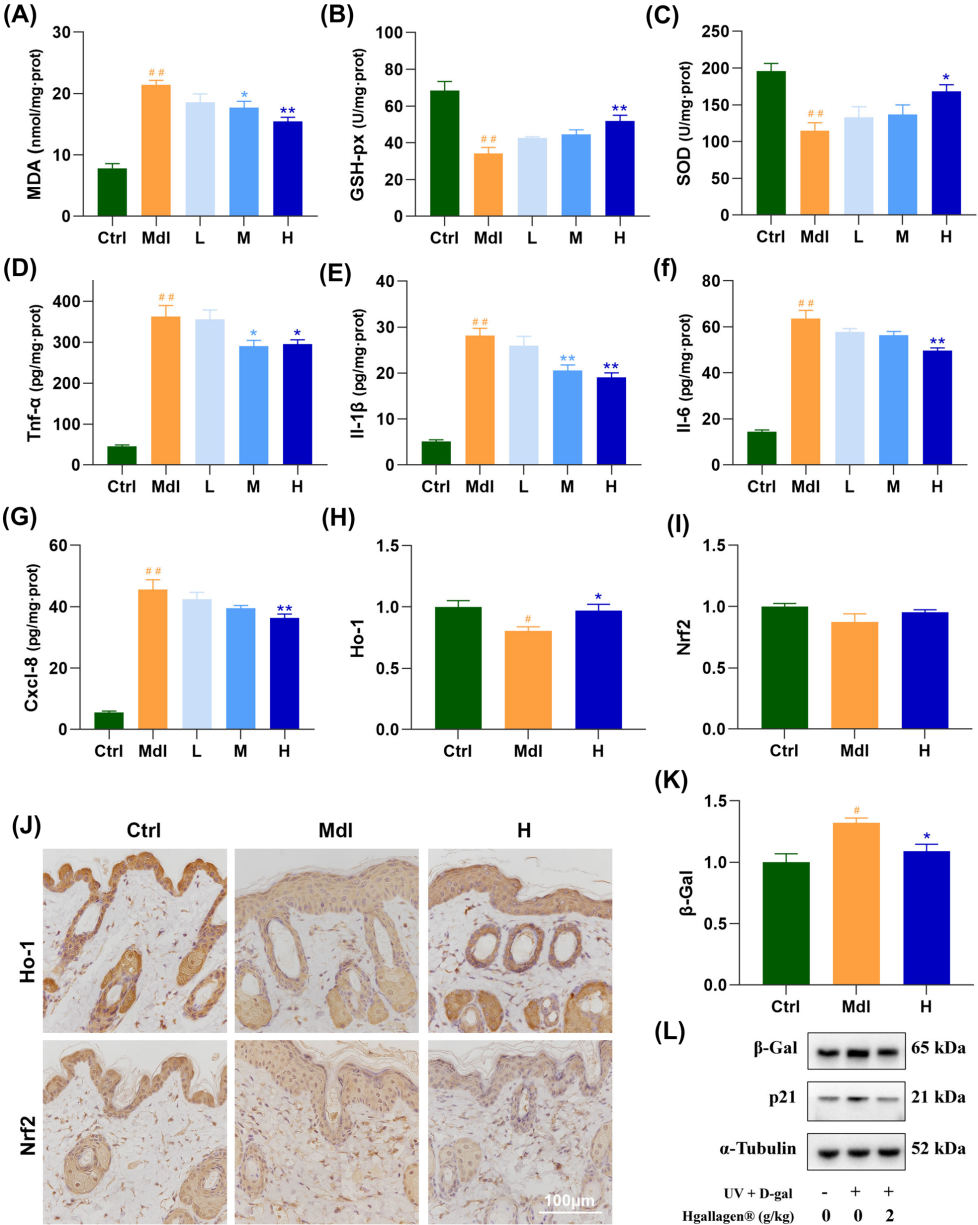
FS-Collagen alleviates UV- and d-gal-induced oxidation, inflammation and cellular senescence. (**A**) MDA content in skin tissue. (**B**) GSH-px activity in skin tissue. (**C**) SOD content in skin tissue. (**D**) TNF-α content in skin tissue. (**E**) IL-β content in skin tissue. (**F**) IL-6 content in skin tissue. (**G**) CXCL-8 content in skin tissue. (**H**–**J**) Immunochemical staining of Ho-1 and Nrf2 in skin tissue. n = 5 in each group. (**K**, **L**) Determination of p21 and β-Gal expressions by western blotting, n = 3. ^#^*p* < 0.05 compared to Ctrl; ^##^*p* < 0.01 compared to Ctrl; *p < 0.05 compared to Mdl; **p < 0.01 compared to Mdl

Besides, as well as reported in other photoaging model, the UV and d-gal treatments significantly induced oxidative stress, leading to increased malondialdehyde (MDA) levels and decreased activities of superoxide dismutase (SOD), Catalase (CAT) and glutathione peroxidase (GSH-px) activity in the Mdl group compared to the Ctrl group. Remarkably, administration of FS-Collagen significantly rescued the activities of SOD, CAT and GSH-px while reducing the MDA levels, suggesting its strong antioxidant effect [Fig. 5(A)–(C)]. Moreover, the expression patterns of Ho-1 and Nrf2 were also determined, and the retrieved Ho-1 expression in the epidermis layer further supports the antioxidant activity of the FS-Collagen [Fig. 5(H)–(J)].

The inflammatory factors including TNF-α, IL-1β, IL-6, CXCL-8 were also uniformly activated in the model group after UV and d-gal treatments, however, FS-Collagen intervention downregulated all of their expressions [Fig. 5(D)–(G)]. More and more researches discovered the association between the inflammation and skin aging in recent years, indicating the anti-inflammation as a new strategy of combating skin aging. Therefore, we directly investigated the senescence-associated markers including p21, p53 and β-Gal in mice of each group. As expected, expression levels of all these markers were induced by UV and d-gal treatment, nonetheless, FS-Collagen administration led to a decrease in their expressions, although the expression levels of p21 and p53 didn’t reach any statistical significance, β-Gal expression level showed an obvious decrease following FS-Collagen intervention [Fig. 5(K), (L); Fig. S1]. However, the inflammation in this model could not represent chronic inflammation in the natural skin aging process, further studies are needed to clarify the role of FS-Collagen against acute and chronic inflammation, and the corresponding effects against aging.

## Supplementary Information

Below is the link to the electronic supplementary material.

Supplementary file1 (DOCX 647 kb)
