# Supplementary material for: Protective effects of a novel FS-Collagen hydrolysates against UV- and d-galactose-induced skin aging
Source: Food Sci Biotechnol. 2024 Aug 7;34(1):257–67. doi: 10.1007/s10068-024-01660-7 (PMC11695533; doi:10.1007/s10068-024-01660-7)
Supplement: Supplementary file 1 — Supplementary file1 (DOCX 647 kb) [file 10068_2024_1660_MOESM1_ESM.docx]

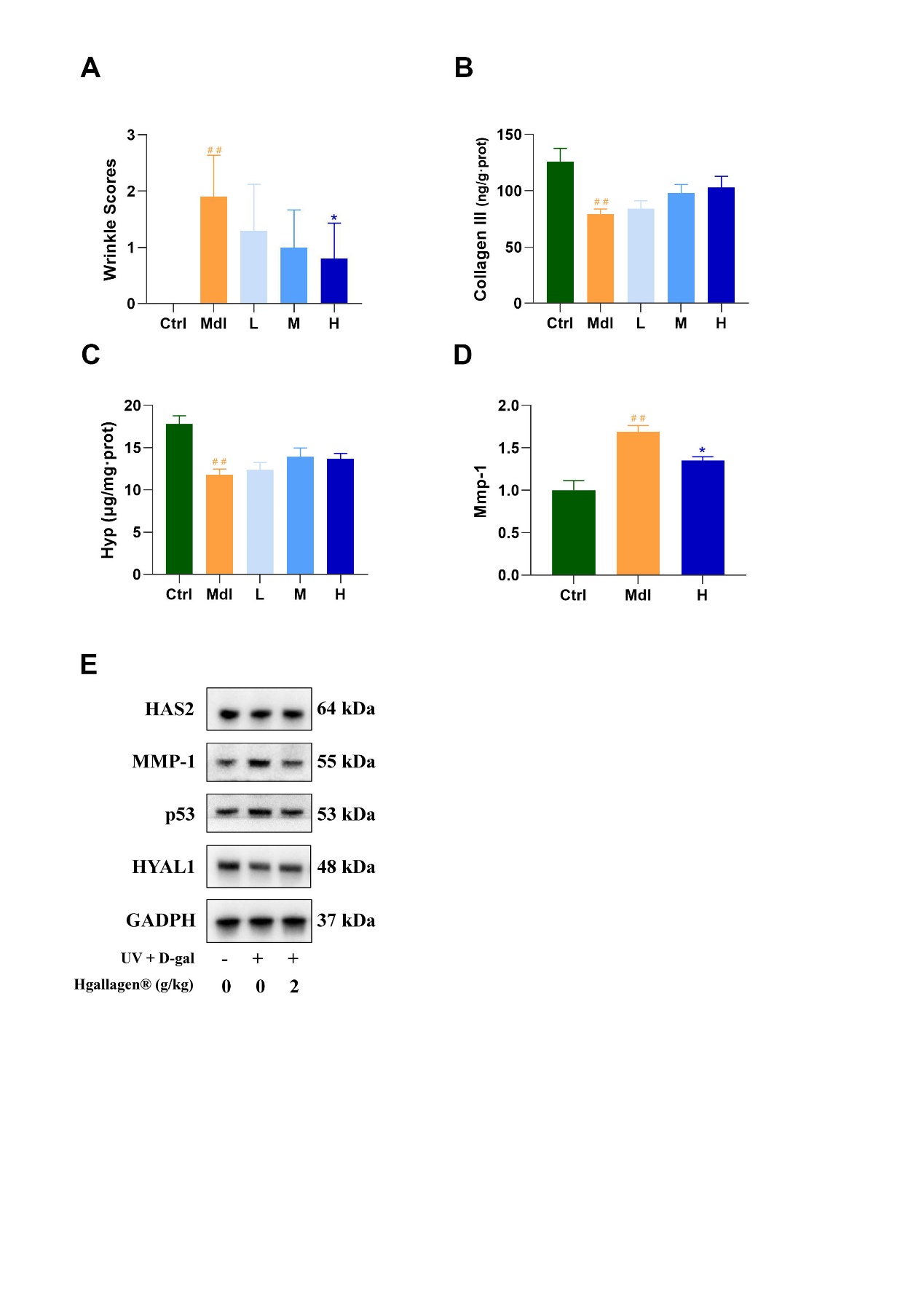


**Figure S1.** Effects of Hgallagen^TM^ on collagen and hyaluronic acid in skin tissue. (A)Wrinkle formation scores (control group is graded with 0 score, representing the smooth skin with almost no wrinkles); (B) Content of collagen III in skin tissue; (C) Content of hydroxyproline in skin tissue; (D) Quantification of Mmp-1 expression; (E) Determination of Has2, Mmp-1, p53, and Hyal1 expression by western blotting. **^# #^** p＜0.01 compared to Ctrl; * p＜0.05 compared to Mdl.


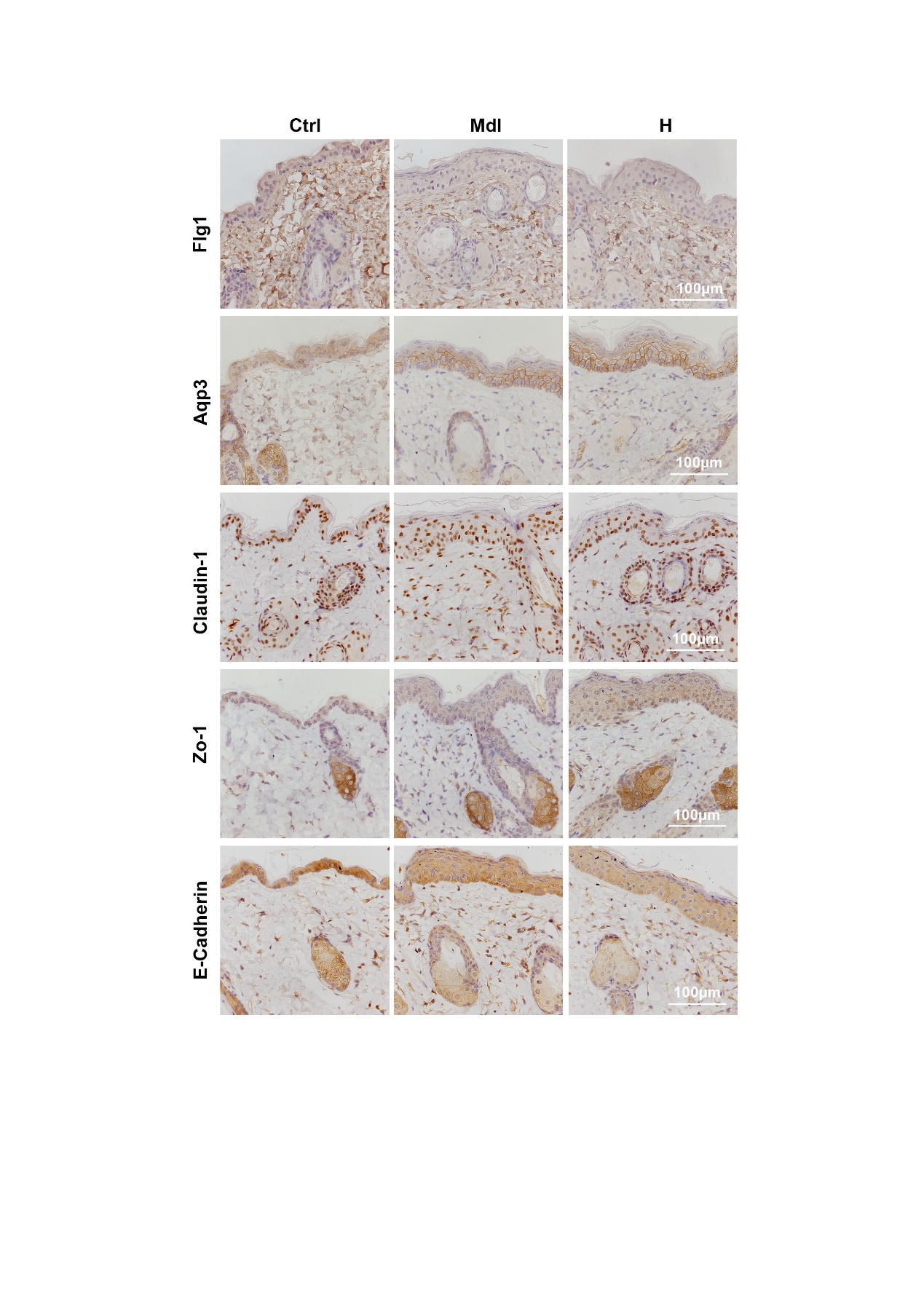


**Figure S2.** Immunochemical staining of Flg1, Aqp3, Claudin-1, Zo-1, E-Cadherin in skin tissue.
